# Localized photodeposition of catalysts using nanophotonic resonances in silicon photocathodes

**DOI:** 10.3762/bjnano.9.198

**Published:** 2018-08-03

**Authors:** Evgenia Kontoleta, Sven H C Askes, Lai-Hung Lai, Erik C Garnett

**Affiliations:** 1 Center for Nanophotonics, AMOLF, Science Park 104, 1098 XG Amsterdam, Netherlands

**Keywords:** catalysts, nanomaterials, nanophotonics, photodeposition, solar fuels

## Abstract

Nanostructured semiconductors feature resonant optical modes that confine light absorption in specific areas called “hot spots”. These areas can be used for localized extraction of the photogenerated charges, which in turn could drive chemical reactions for synthesis of catalytic materials. In this work, we use these nanophotonic hot spots in vertical silicon nanowires to locally deposit platinum nanoparticles in a photo-electrochemical system. The tapering angle of the silicon nanowires as well as the excitation wavelength are used to control the location of the hot spots together with the deposition sites of the platinum catalyst. A combination of finite difference time domain (FDTD) simulations with scanning electron microscopy image analysis showed a reasonable correlation between the simulated hot spots and the actual experimental localization and quantity of platinum atoms. This nanophotonic approach of driving chemical reactions at the nanoscale using the optical properties of the photo-electrode, can be very promising for the design of lithography-free and efficient hierarchical nanostructures for the generation of solar fuels.

## Introduction

The relentless rise of CO_2_ levels in the atmosphere as well as the growth of the world population remind us of the importance of finding new, clean pathways to cover our energy needs. Fuel generation from renewable energy resources could be one of the “clean” approaches for meeting our energy requirements. Although, sunlight is the most abundant source of green energy, its long-term storage is required, due to daily and yearly fluctuations. The most promising medium for stable, high-density storage is in the form of chemical energy, such as H_2_ or organic compounds, by using photochemical fuel generators [1–4].

In the center of a photochemical fuel generator are the photo-electrodes, where light absorption and conversion to chemical energy take place. The photo-electrodes are in contact with an electrolyte that is the primary source of fuel together with the sunlight. In such a system, light absorption by the electrodes leads to the creation of electron–hole pairs, which after their separation participate in chemical reactions in the electrolyte to make fuels. One example is water splitting for H_2_ generation [5–6]. Carefully designed photo-electrodes are necessary for low cost and high efficiency, which are both needed to make solar fuels competitive with fossil fuels as an energy carrier. Nanostructuring the main photoactive material, e.g., a semiconductor, has proven to be a promising method for increasing the efficiency of solar fuel generation [7–8]. The higher surface to volume ratio in nanostructured semiconductors ensures the use of less material, reduces the requirements on current density and often increases light absorption. This increased light absorption comes from optical resonances in nanomaterials, which have been studied extensively in both metallic (plasmonic) and dielectric material systems [9–13]. One hallmark of resonant absorption is the appearance of localized “hot spots”. In particular, semiconducting nanostructures can sustain Mie-like leaky modes due to their high refractive index and the occurrence of multiple internal light reflections from the boundaries of the structure [9,13]. However, in vertical nanowires under normal-incidence illumination, Mie modes cannot be excited and instead coupling to waveguide modes (e.g., the HE_11_) and subsequent Fabry–Perot cavity interference play the dominant role in creating these hot spots [14–15]. The highly concentrated electric fields at the hot spots lead to elevated concentrations of photogenerated charge carriers that can be used to drive solar fuel reactions [16–19]. Additionally, photochemical fuel generators require a catalyst, such as platinum, to lower the overpotential to drive the chemical reaction [2,7,20–24]. The catalyst would be ideally located at the semiconductor–solution interface, directly at the location of the hot spots.

Placing the catalyst exclusively at the hot spots would reduce both the catalyst loading (lowering the cost) and the average time between charge generation and chemical reaction (increasing the efficiency). However, current catalysts are simply randomly placed on semiconductor photo-electrodes with an optimized average density [20,24–25]. Photodeposition of the catalytic material with photogenerated charges from excited semiconductors has been also achieved but without a good control over the deposition sites [26–31]. An exception is the work of Li et al. [27], where charge separation was achieved at different crystal facets of BiVO_4_ nanocrystals for selective photodeposition of metal and metal oxide catalytic nanoparticles. Nevertheless, this method for the moment is limited to this specific material and structure.

Here we present a different approach in which localized nanophotonic resonances in semiconductors are used to place catalyst particles exactly where they are needed. We show that the location of catalyst deposition on vertical silicon nanowires can be tuned by adjusting their shape (tapering angle) or changing the excitation wavelength. The experimentally observed deposition profiles match reasonably well with optical simulations of the photogenerated charge carrier distribution for each shape and wavelength. Most notably, deposition profiles far from those expected from a simple Beer–Lambert law have been obtained, in contrast to previous related work on silicon microwires [32–33]. Our results provide the first step for rationally designed catalyst positioning using the underlying resonant properties of nanoscale photocatalysts, tunable simply by altering the shape, size or excitation wavelength. The extensive literature on such nanophotonic tuning makes this an exciting approach for lithography-free nanoscale control over catalyst positioning [34–38].

**Figure 1:**
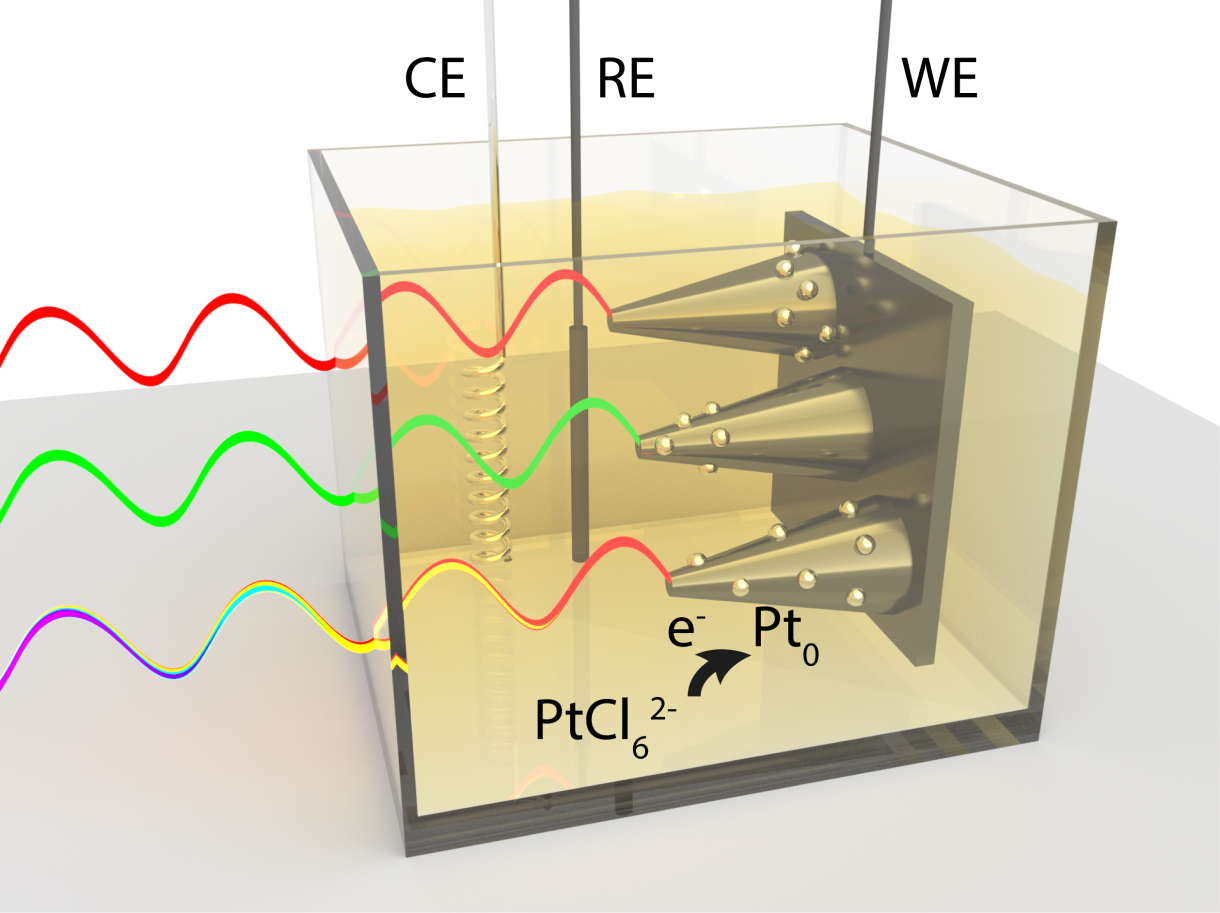
Schematic illustration of the photo-electrochemical deposition of metallic Pt on silicon nanostructures from hexachloroplatinate (PtCl_6_^2−^) in a three-electrode photo-electrochemical cell with counter electrode (CE), reference electrode (RE) and working electrode (WE). The location of catalyst deposition can be tuned by adjusting the excitation wavelength from red to green to white, or (not shown) the nanostructure shape.

We have chosen vertical silicon nanowires as a model nanophotonic system because of their ease of fabrication, known optical constants and broad spectral absorption range. In presence of a Pt-catalyst precursor (H_2_PtCl_6_) in a three-electrode photo-electrochemical system (Figure 1), photogenerated electrons reach the surface of the silicon nanowires, reducing the precursor to form metallic platinum nanoparticles (Pt(0)). The position of the Pt deposition can be controlled by adjusting the tapering angle or the incident wavelength. The platinum photodeposition results are observed with a scanning electron microscope (SEM) and compared with the output of finite difference time domain (FDTD) simulations of the density distribution of the photogenerated carriers within the silicon nanostructures.

## Results and Discussion

### Fabrication of silicon nanostructures and calculation of their optical modes

**Figure 2:**
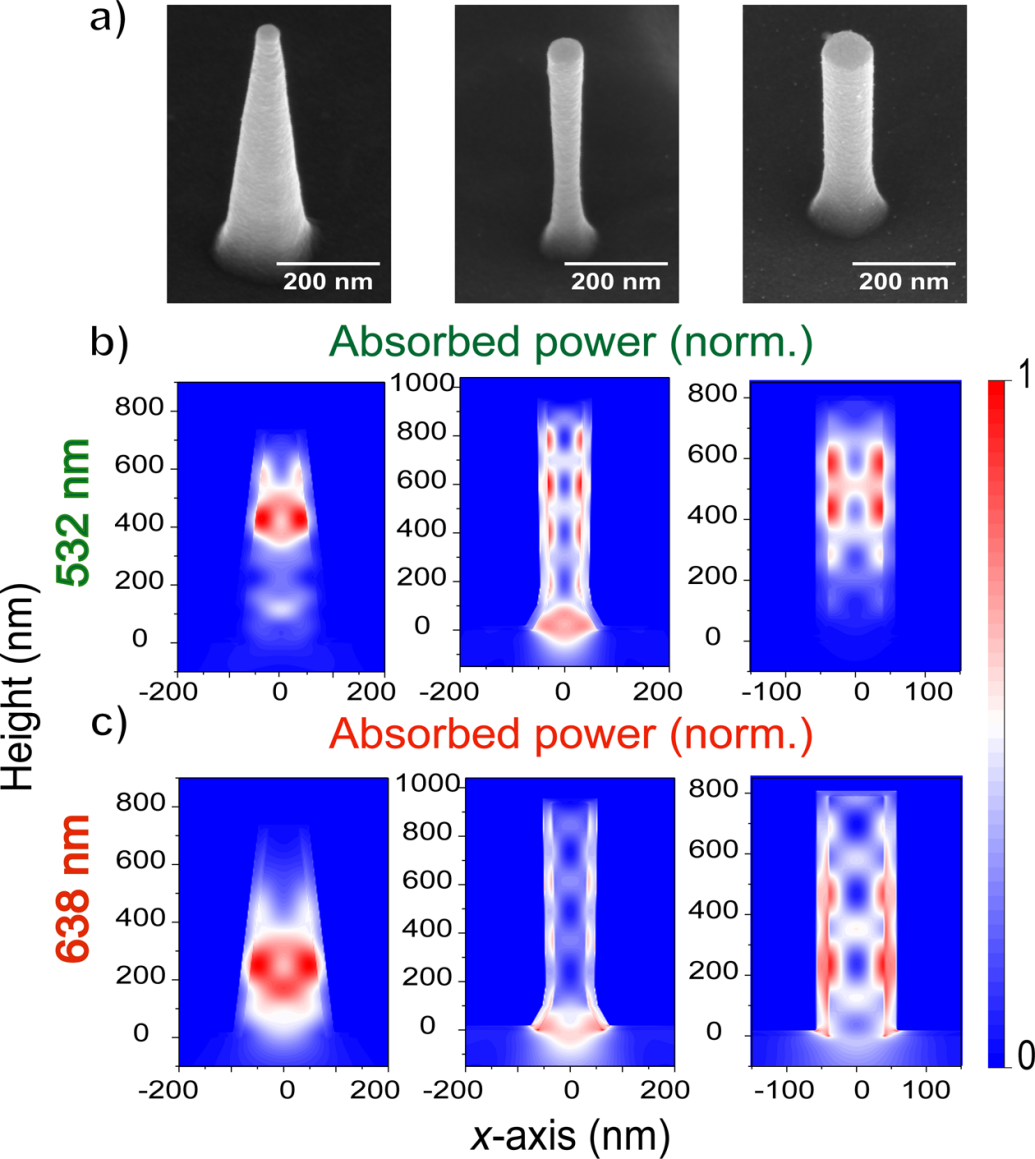
(a) SEM images of a silicon nanocone (left), an inverted nanocone (middle) and a nanowire (right) coated with an 18 nm TiO_2_ layer. The tapering angle was controlled by varying the Cl_2_ and HBr/O_2_ flow rates during plasma etching. (b, c) FDTD simulations of absorbed power in each nanostructure at (b) 532 nm and (c) 638 nm normalized to the maximum value.

Silicon nanostructures were fabricated by etching a p-type silicon substrate using a combination of Cl_2_ and HBr/O_2_ plasmas with 110 nm diameter SiO_2_ spheres being used as etch mask. Tuning the ratio of the HBr/O_2_ plasmas allowed for vertical nanowires with variable sidewall tapering angle (see details in Experimental section and Figure S1, Supporting Information File 1). Vertical nanocones (height ≈ 720 nm, top diameter ≈ 60 nm, bottom diameter ≈ 160 nm, angle ≈ 15°), inverted nanocones (height ≈ 1 μm, top diameter ≈ 70 nm, base diameter ≈ 120 nm and smallest diameter ≈ 60 nm) and nanowires (height ≈ 790 nm, diameter ≈ 80 nm) were fabricated here and subsequently used to tune the distribution of photogenerated carriers (Figure 2). An 18 nm amorphous TiO_2_ layer was conformally deposited on the silicon nanostructures by using atomic layer deposition (ALD). This layer assists with charge separation, stabilizes the silicon surface and helps to passivate trap states, leading to well-known improvements in photo-electrochemical performance [39–41]. The amorphous TiO_2_ layer was further annealed at 350 °C for 3 h to form crystalline anatase TiO_2_, which led to an improved performance. The final TiO_2_ layers were characterized with X-ray diffraction (XRD) (Figure S2, Supporting Information File 1) and ellipsometry (Figure S3, Supporting Information File 1) to verify their quality. Both the XRD pattern and optical constants (*n* and *k* values) matched the literature values for thin anatase TiO_2_ films [42].

The photocarrier density distribution under monochromatic illumination (532 or 638 nm) in the Si–TiO_2_ nanostructures was simulated using the FDTD method. It was assumed that every absorbed photon was converted to an electron–hole pair and only the optical effects were taken into account in the simulations. The dimensions of the average silicon nanostructures extracted from SEM images were used for the simulations, while the *n* and *k* values measured with ellipsometry were used for the TiO_2_ coating. Every structure was simulated on a thick silicon substrate, also coated with 18 nm TiO_2_, and the surrounding refractive index was set to 1.33 to resemble the aqueous conditions of the reaction environment. The simulations show the cross-sectional absorbed power profiles (normalized to the maximum value per plot) of the three different silicon nanostructures, for excitation at 532 nm (Figure 2b) and 638 nm (Figure 2c). The location of the hot spots depends on the excitation wavelength and the shape of the nanostructure. Silicon nanocones confine light mostly at the top of the structure at 532 nm (Figure 2b) in contrast to an excitation at 638 nm, where most of the light is absorbed at the bottom of the cone (Figure 2c). In the case of inverted nanocones, light is concentrated primarily at the bottom for both wavelengths, although at 532 nm there are also additional hot spots along the height. In silicon nanowires, hot spots are present at the top and the middle of the structure for 532 nm but for excitation at 638 nm, more hot spots appear. Overall, the results of these simulations confirm the presence of distinct hot spots in the nanostructures and enable us to investigate whether the simulated hot spots match the location of the deposited catalytic material after illumination.

### Photodeposition of platinum

**Figure 3:**
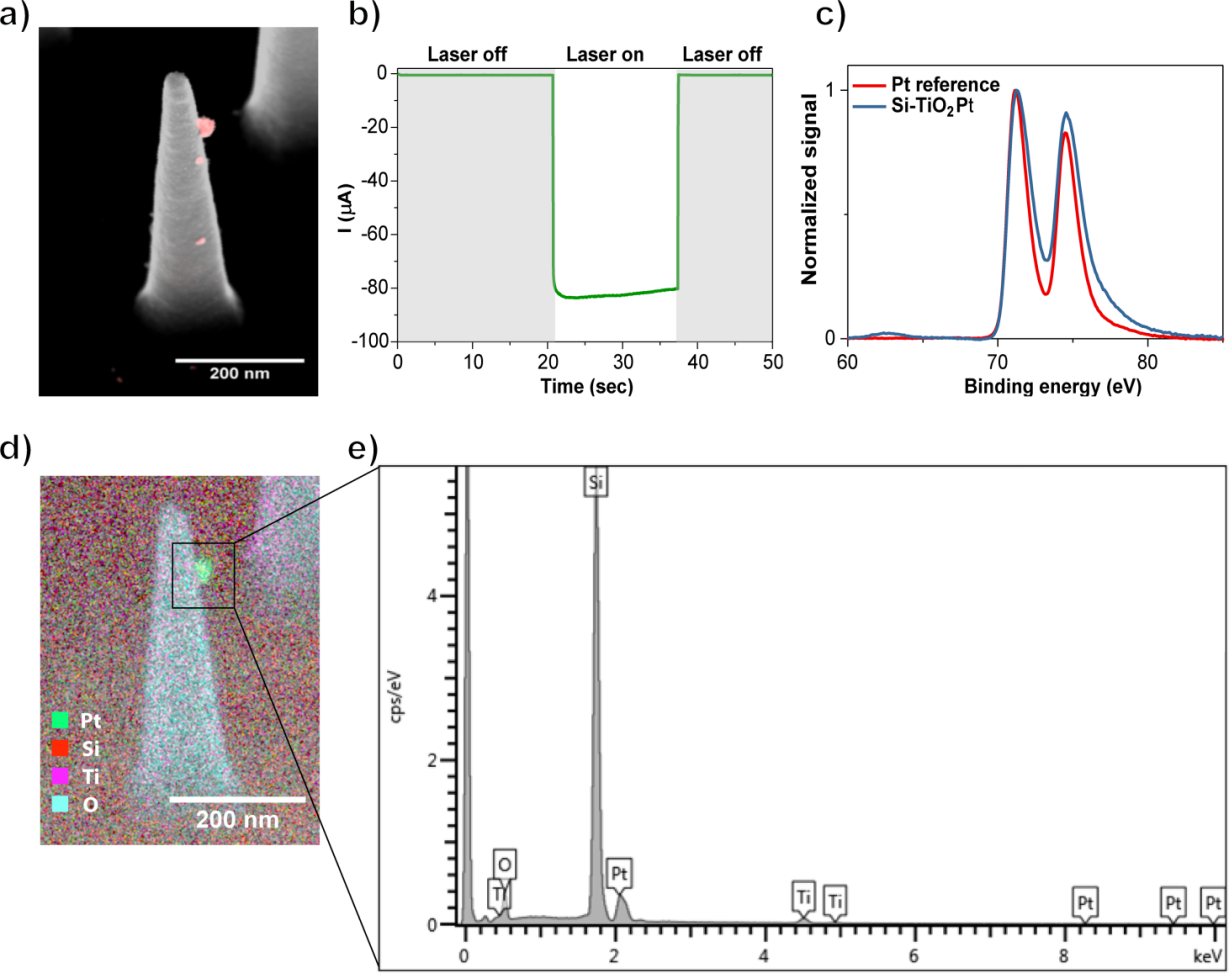
a) An overlay image of a backscattered electron (red; in-lens mirror detector) and secondary electron (grey; through-the-lens detector) SEM image of a silicon nanocone after photo-electrodeposition of platinum. b) Current as a function of the time during a photo-electrodeposition experiment of silicon nanocones excited at 532 nm (laser on), at −0.8 V applied potential in an aqueous solution of H_2_PtCl_6_ (pH 11). c) X-ray photoemission spectrum of photo-electrodeposited platinum on silicon nanostructures (blue) compared with the spectrum of a metallic Pt reference material (red). d) EDS elemental map where each color indicates a different element: Pt (green), Si (red), Ti (purple) and O (cyan). e) Elemental map retrieved from an individual deposited particle (100 pA beam current, 10 kV acceleration beam voltage).

A three-electrode photo-electrochemical cell, electrically connected with a potentiostat, was used for deposition of the platinum catalyst on the nanostructures. The sample served as the working electrode (WE) with a platinum wire counter electrode (CE) and Ag/AgCl reference electrode (RE) (Figure 1). During a typical photo-electrodeposition experiment, the sample was mounted in direct contact with a Pt-precursor electrolyte (4 mM H_2_PtCl_6_, pH 11) and the current flow to the working electrode was recorded as a function of time at a constant electrochemical potential, i.e., in the chronoamperometry mode. The samples had an open-circuit voltage potential of around −0.1 V (vs Ag/AgCl) and were biased by 700 mV to a more reducing potential of −0.8 V (vs Ag/AgCl) during deposition, to efficiently extract the photogenerated charges from the Si nanostructures into the electrolyte and enhance the kinetics of the reaction. The flat-band potential of TiO_2_ at pH 11 is above the conduction band edge of p-type silicon, so TiO_2_ acts as an electron blocking layer here [5,43–44]. Therefore, the presence of TiO_2_ offers a control over the potential we could apply to selectively promote photodeposition while avoiding electrodeposition. In the absence of a TiO_2_ layer the recorded dark current is much higher than the corresponding photocurrent (Figure S4a, Supporting Information File 1), which means that the electrons reaching the electrolyte by biasing the samples dominate over the photogenerated ones. SEM images (Figure S5, Supporting Information File 1) show the homogenous formation of platinum nanoparticles both on the Si nanostructures and on the substrate, when the samples were illuminated without the TiO_2_ layer but still under biased conditions. The final potential value (−0.8 V) for photo-electrodeposition of platinum nanoparticles in the presence of a TiO_2_ layer was chosen because it yields a high current ratio between illumination and dark conditions (Figure S6, Supporting Information File 1). Even more negative potentials than −0.8 V could be used here, but it was not necessary since the kinetics of the reaction were fast enough to drive the photo-electrodeposition in a few seconds. Typically, the current was 75–200 times greater with illumination than without. As shown in Figure 3b, during the first 20 s of a typical photo-electrodeposition experiment using 532 nm light, the laser beam was blocked and the current was recorded. As soon as the laser beam hit the sample, a current increase was observed due to the contribution of the photogenerated charges. After an electrical charge of around 1.35 mC was passed to the illuminated sample, the laser beam was blocked again and the measurement was stopped.

The area of the laser beam (0.06 mm^2^ for 532 nm and 0.35 mm^2^ for 638 nm) was much smaller than the surface of the samples in contact with the electrolyte (0.3 cm^2^), which allowed straightforward identification of the illuminated area and discrimination of platinum deposition under light and dark conditions in the same experiment (Figure S7, Supporting Information File 1). SEM images (Figure 3a and Figure S8, Supporting Information File 1) taken from the illuminated region revealed the presence of new nanoparticles on the silicon nanostructures and substrate. These were not observed far from the illuminated region (Figure S9, Supporting Information File 1), which confirmed that the irradiation had caused the formation of nanoparticles. Energy-dispersive X-ray spectrometry (EDS) mapping clearly confirmed the presence of platinum, when an individual newly formed particle was analyzed (Figure 3d and Figure 3e). Furthermore, the oxidation state of platinum was investigated with X-ray photo-electron spectroscopy (XPS) on a sample with a higher amount of photo-electrodeposited platinum (ca. 2 mC). The observed platinum 4f_7/2_ and 4f_5/2_ binding energy peaks corresponded very well to those of a metallic Pt reference material (Figure 3c). Overall, these results demonstrate that light can be used as an external stimulus for the formation of catalytic Pt(0) material on Si nanostructures.

### Correlation of hot spots and Pt deposition sites

**Figure 4:**
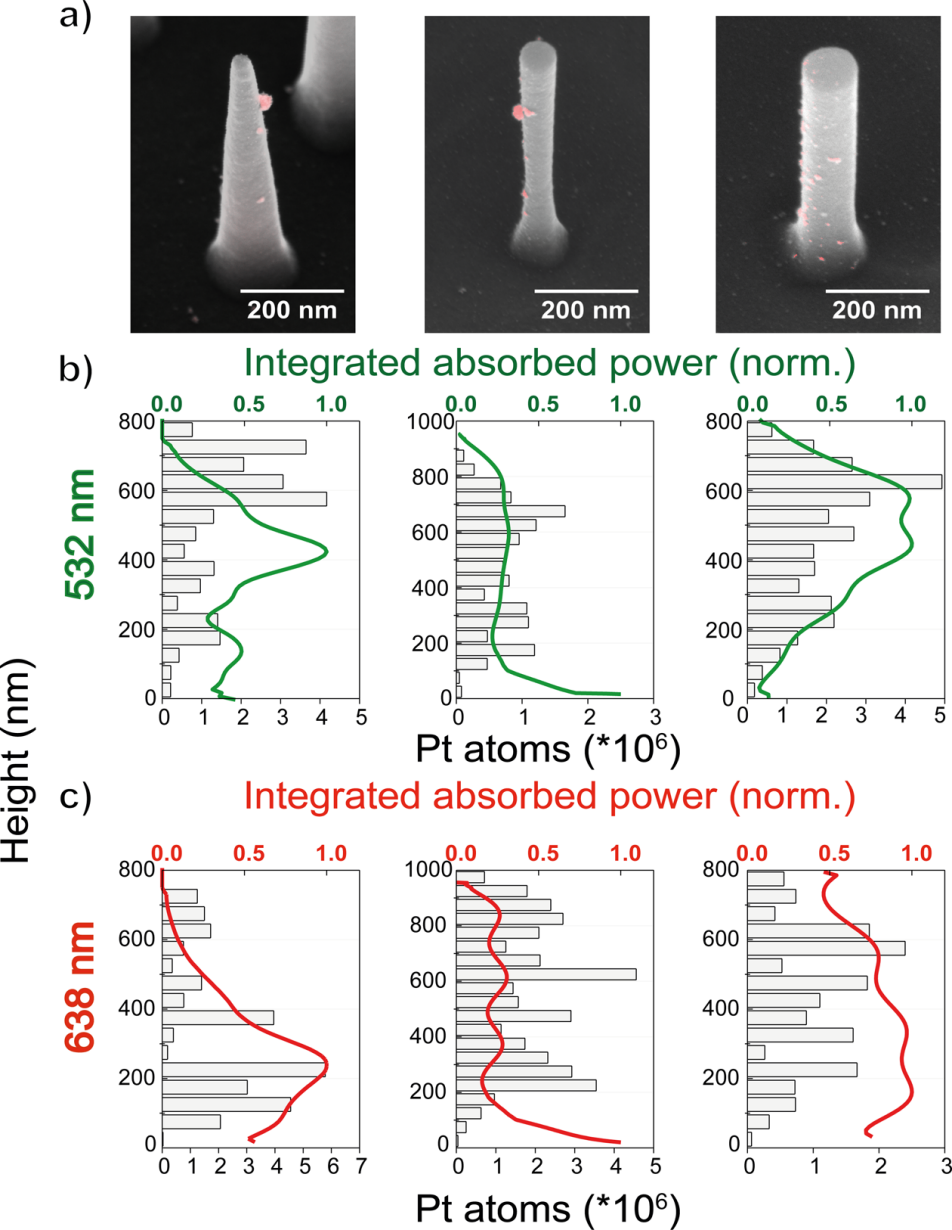
(a) Overlay images of backscattered electron (red) and secondary-electron (grey) SEM images after photo-electrodeposition of platinum on a silicon nanocone (left), inverted nanocone (middle), and nanowire (right). (b, c) Total amount of platinum atoms deposited (grey bars) along the height of each silicon structure for excitations at (b) 532 nm and (c) 638 nm. Each graph includes the accumulated values of 100 structures. Green and red solid lines correspond to the integrated absorbed power (normalized to the maximum value) as a function of the height at 532 nm and 638 nm, respectively.

Next, a comparison was made between the Pt deposition sites and the simulated optical hot spots of the Si nanostructures with an SEM image analysis approach, as follows: First, preliminary chronoamperometric experiments were conducted to indicate the conditions in which we could easily identify the location of the platinum particles on each nanostructure without the total overgrowth of the latter. A total amount of around 1 mC was needed to obtain well separated Pt particles with a diameter of 11 nm, which typically corresponded to 15–20 s of illumination at 532 or 638 nm with a light intensity of 1.2 W/cm^2^ or 0.35 W/cm^2^, respectively. The size of the deposited platinum nanoparticles was selected only for particle identification purposes and further optimization of the photo-electrodeposition process is necessary for the fabrication of efficient photocatalytic samples. For each Si nanostructure morphology, overlays of secondary-electron and backscattered-electron (collected with an in-lens mirror detector) SEM images were acquired. This overlay method facilitates the identification of platinum nanoparticles based on the high electron backscattering efficiency of this heavy element (Figure 4a and Figures S10–S12, Supporting Information File 1). Images were collected from 100 individual nanostructures of each morphology while exclusively considering structures with dimensions within half a standard deviation of the average structure. Furthermore, platinum particles with a diameter below 6 nm were excluded, as they could also originate from electrodeposition (Figure S9, Supporting Information File 1). The volume of each Pt nanoparticle was estimated and converted to the corresponding number of platinum atoms. Finally, histograms were made to visualize the deposited amount of Pt as a function of the Si nanostructure height (Figure 4b and Figure 4c). The results are presented together with the simulated integrated absorbed power (normalized to the maximum value per plot) along the height of every structure at 532 and 638 nm.

Comparison of the platinum deposition distribution on the silicon nanostructures and the integrated absorbed power profiles reveals that they match reasonably. Specifically, for silicon nanocones a correlation of the platinum deposition sites and the optical modes is shown for both excitation wavelengths with some deviations. At 532 nm, the two peaks of the platinum distribution are slightly shifted towards larger heights, while at 638 nm deposition of platinum is also observed in locations not expected from the absorbed power simulations, i.e., at the top of the nanostructure. In the case of inverted nanocones, the platinum deposition profiles seem to follow the profiles of the integrated absorbed power. However, simulations showed that most of the light is absorbed at the bottom of the nanostructure, where no platinum is observed in the experiments. In contrast, deposition of the catalytic material occurs primarily at a height of around 200–300 nm from the bottom. This discrepancy may be explained by the fact that both nanocones and inverted nanocones exhibit structural diameter differences along their height, which could lead to differences in carrier collection efficiency if the diffusion length is of the order of, or smaller than, the diameter. Such variations in carrier collection efficiency would naturally alter the final deposition distribution in a manner qualitatively consistent with our observations. Finally, silicon nanowires excited at 532 nm concentrate the incident light mostly at the top or the middle of the structure, corresponding well with the platinum deposition analysis. For 638 nm excitation, the same structures exhibited multiple deposition sites along their height, which is also correlated to the integrated absorbed power peaks.

Instead of the formation of new small particles of platinum on the silicon nanostructures, overgrowth of the already deposited ones was noticed from the SEM images (Figures S10–S12, Supporting Information File 1). This effect could be explained by the fact that platinum nanoparticles act as electron-trapping centers on the surface of TiO_2_ [45–46]. After the formation of the very first platinum nanoparticles, photogenerated electrons from the silicon nanostructures are transferred to TiO_2_ and in sequence to the already formed platinum. The Schottky barrier between TiO_2_ and platinum nanoparticles does not allow for a “back” transfer of electrons. Hence, charge separation is promoted, which allows further reduction of hexachloroplatinate to Pt(0) on one of the existing platinum nanoparticles rather than in new locations. As a result, the initial platinum nanoparticle formation may alter the final deposition profile from the simulated one by prohibiting the deposition at other parts of the nanostructrure. As mentioned earlier, an external electric field is applied to the samples for more efficient extraction of the photogenerated charges. This electric field is not taken into account in the simulated distribution of the charges along the height of the Si nanostructures (Figure 2), and this is another factor that could affect the localization of the photo-electrodeposition. The platinum deposition could also be broadened compared to the simulated profile due to our method of measuring the height of each particle, which extracts 3D distances from a 2D image. Noise could also be introduced by the TiO_2_ layer itself. Although TiO_2_ has a shorter electron diffusion length compared to silicon [47–48], the TiO_2_ surface could also have randomly distributed surface sites with higher catalytic activity, leading to preferential deposition, or traps that capture carriers preventing deposition.

## Conclusion

We show that the optical modes of silicon nanostructures can be used for lithography-free patterning of catalytic nanoparticles. Tuning of the photo-electrochemical formation of platinum nanoparticles along the height of silicon nanostructures was achieved by changing either the shape (tapering angle) of the silicon nanostructures or the excitation wavelength (red or green light). This method utilizing the optical modes of semiconducting nanostructures to pattern catalytic materials with nanoscale control can be a very promising method for an easy and low-cost fabrication of efficient photo-electrodes. It provides a lot of flexibility on the materials involved and on the design of the final structure. Further research should be focused on improving the positioning precision and implementing the approach in a state-of-the art photo-electrode/catalyst system in order to demonstrate the potential for solar fuel production enhancement.

## Experimental

### General

Chemicals were purchased from major chemical suppliers and used as received. Scanning electron microscopy (SEM) was performed on a FEI Verios 460 with a typical acceleration beam voltage of 5 kV and 100 pA beam current. Secondary-electron images were collected with a through-the-lens detector (TLD) and backscattered-electron images were collected with an in-lens mirror detector. Energy dispersive X-ray spectrometry (EDS) was performed with an Oxford Instruments device with an acceleration beam voltage of 10 kV and beam current of 100 pA. X-ray diffraction was done with a Bruker D2 Phaser with Cu Kα radiation (λ = 1.5418 Å).

### Simulations

Lumerical FDTD Solutions was used for simulations of single silicon nanostructures on a 3.5 × 3.5 × 2 μm silicon substrate. Absorbed power simulations were conducted with an 18 nm TiO_2_ layer, with refractive index values (*n* and *k*) retrieved from ellipsometry (Figure S3, Supporting Information File 1). An example of the simulation environment can be found in Figure S13 (Supporting Information File 1) in which the case of inverted nanocones is presented. The structures were excited with a plane wave source with wavelengths of 400–1100 nm and the absorbed power was retrieved from an absorption per unit volume monitor with wavelength selection option. The refractive index of the surrounding medium was set to 1.33. The mesh size in the FDTD simulations was equal to 2 × 2 × 2 nm for all the structures.

### Fabrication of silicon nanostructures

Silicon p-type samples (Active Business Company GmbH, <100> orientation ) 12 × 12 mm, with 1–10 Ω·cm resistivity, were used as substrates for the fabrication of the three different types of silicon nanostructures. First, the samples were cleaned with soap and consecutively rinsed with copious amounts of water, acetone and isopropanol. After that, the samples were submerged in hot piranha solution (120 °C, 3:1 concentrated H_2_SO_4_/30% H_2_O_2_) for 20 min and rinsed with deionized water. Then 2–3 μL of 110 nm diameter SiO_2_ spheres dispersed in ethanol were drop-cast on the clean silicon samples and annealed for 1 min at 60 °C on a hot plate. The samples were etched with a combination of plasmas (PlasmaPro 100 Cobra ICP Etch). First Cl_2_ (20 sec, 50 sccm, HF forward power 40 W, 7 mTorr) was used for removal of the native oxide and then HBr/O_2_ (5 min for nanocones and 11 min for nanowires and inverted nanocones, HF forward power 30 W, 7 mTorr) was used for etching the silicon to the desired structures. Before the etching steps an oxygen cleaning step was used (1 min and 30 sec, 50 sccm O_2_, HF forward power 60 W, ICP forward power 100 W, 6 mTorr). The temperature used for all the steps of the plasma etching was 20 °C. The ratio of HBr/O_2_ was very crucial for the control of the shape of the silicon structures. For the nanocones a ratio of 48.2:1.8 sccm (HBr/O_2_) was chosen, 49.5:0.5 for inverted nanocones and 49:1 for stand-up nanowires. The ICP forward power during Cl_2_ and HBr/O_2_ etching was 750 W for silicon nanocones and nanowires, and 600 W for inverted nanocones. After etching, the samples were treated with 7 vol % HF solution for the removal of SiO_2_ formed during the etching procedure, rinsed with water, dipped in hot piranha solution for 20 min and rinsed one more time with water. The last step (hot piranha solution) proved necessary to obtain a smooth coating of the structures with TiO_2_, probably due to the increase of the hydrophilicity.

### Formation of TiO_2_ using atomic layer deposition

A custom-built atomic layer deposition system was used for the deposition of thin and compact TiO_2_ layers on the silicon nanostructures. For 18 nm TiO_2_ layers, subsequent injection of MilliQ water (18.2 MΩ·cm) and 99.995 % TiCl_4_ (for 10 ms each) took place in a vacuum chamber with a delay of 18 s between each injection. The samples were heated by a copper stage at 100 °C. The base pressure of the system was below 5·10^−2^ mbar. The pressure during deposition was adjusted to 1.1 mbar using an N_2_ purging flow to remove the formed gases and excess precursors. Post-annealing of the samples in a tube oven, in air, at 350 °C for 3 h with a ramp of 11 °C/min was needed for the formation of anatase TiO_2_ (Figure S2, Supporting Information File 1).

### Photo-electrochemical deposition

For the deposition of platinum nanoparticles, a photo-electrochemical cell (Zahner Scientific Instruments, PECC-1, slightly modified) made from Teflon was used (Figure S14, Supporting Information File 1). The cell has three inputs for the three different electrodes (working, reference and counter). Only a small area (0.3 cm^2^) of the working electrode (i.e., the sample) was in contact with the electrolyte, which was illuminated through a quartz window. The electrolyte consisted of an aqueous solution of chloroplatinic acid (4 mM) and Na_2_SO_4_ (0.1 M), with the pH value adjusted to 11 with 2 M NaOH. The back contact of the sample consisted of 4 nm of chromium and 50 nm of gold deposited with a double-target sputter coater (Leica EM ACE600). The electrical connections of the sample with the potentiostat (BioLogic Science Instruments, SP-200) were made using conductive aluminum tape (Advance Tapes AT521) adhered to the back metal contact of the sample, which was not in contact with the electrolyte.

### X-ray photoemission spectroscopy (XPS)

X-ray photoemission spectroscopy (XPS) was performed in a custom-built ultrahigh-vacuum chamber, operating at a base pressure below 5 × 10^−9^ mbar. A XM1200 monochromatic X-ray source (Al Kα line, Scienta Omicron) was used for X-ray excitation of the sample under a 45° angle. Photoemitted electrons were collected using a HIPP-3 analyzer (Scienta Omicron). A polished platinum pellet (99.99%, Kurt J. Lesker Company) was used for acquiring a Pt reference spectrum. Spectra were charge-corrected using the binding energy of C 1s (284.8 eV).

## Supporting Information

Schematic description of the experimental process step by step for the Si nanostructures fabrication; XRD and ellipsometry data of the TiO_2_ layer; chronoamperometry measurements of Si nanocones with and without TiO_2_ layer; SEM image of Si nanocone after illumination without the TiO_2_ layer; current-vs-potential measurement on silicon nanocones; SEM images in and out of the illumination spot and of the illumination spot itself; representation of the photo-electrochemical cell; schematic diagram of the FDTD simulations and SEM images of more silicon nanostructures after photo-electrodeposition of platinum for verification of the effect.

File 1 Additional experimental data.
