# Supplementary material for: Localized photodeposition of catalysts using nanophotonic resonances in silicon photocathodes
Source: Beilstein J Nanotechnol. 2018 Aug 3;9:2097–105. doi: 10.3762/bjnano.9.198 (PMC6122171; doi:10.3762/bjnano.9.198)
Supplement: File 1 — Additional experimental data. [file Beilstein_J_Nanotechnol-09-2097-s001.pdf]

# Supporting Information

for

# Localized photodeposition of catalysts using nanophotonic resonances in silicon photocathodes

Evgenia Kontoleta, Sven H. C. Askes, Lai-Hung Lai and Erik C. Garnett*

Address: Center for Nanophotonics, AMOLF, Science Park 104, 1098 XG Amsterdam, Netherlands.

Email: Erik C. Garnett* - E.Garnett@amolf.nl

*Corresponding author

## Additional experimental data

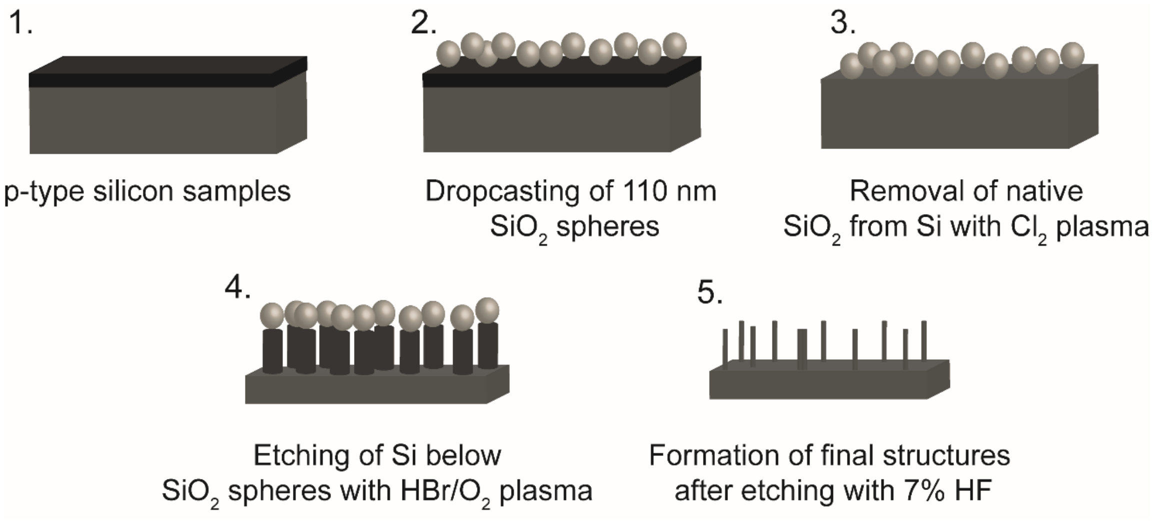


**Figure S1:** Description of the process steps for the preparation of silicon nanostructures with plasma etching.

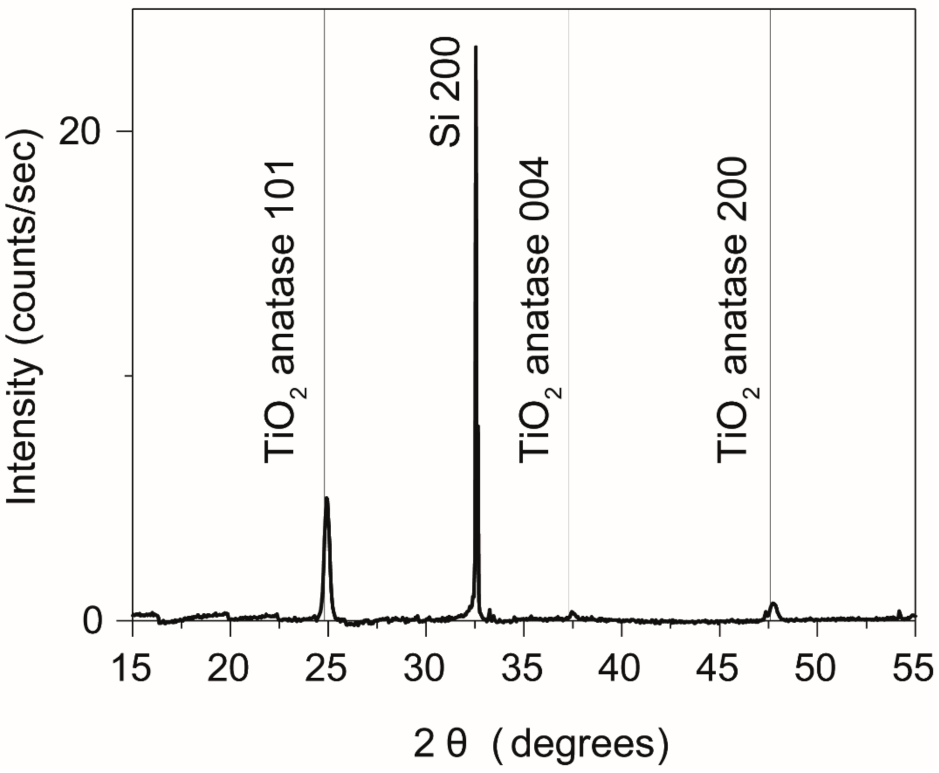


**Figure S2:** XRD data of an annealed $TiO_2$ layer of around 54 nm on a p-type silicon substrate. Anatase reference peaks (JCPDS Card no. 21-1272) shown as grey lines.

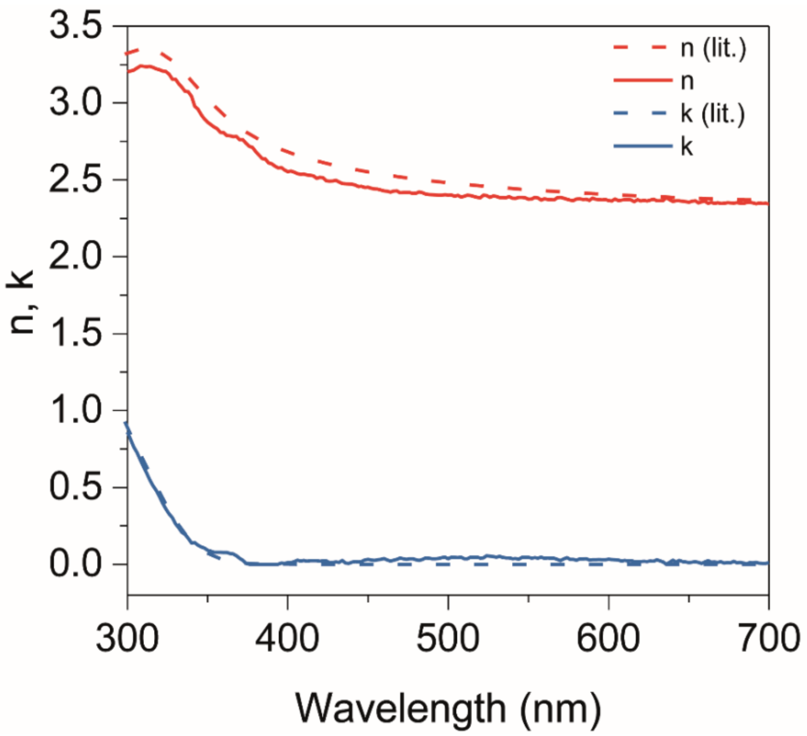


**Figure S3:** Optical constants retrieved from ellipsometry measurements of 18 nm $TiO_2$ prepared with ALD on a flat p-type silicon substrate and subsequent annealing for 3 h at 350 °C [1].

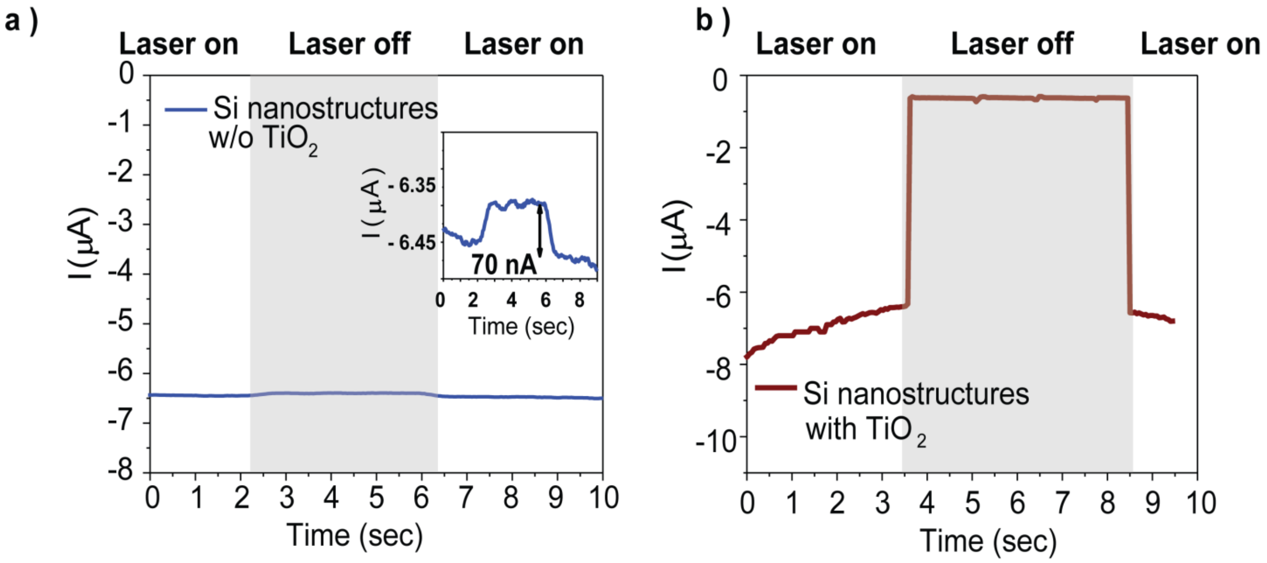


**Figure S4:** Chronoamperometry measurements of a silicon nanocone sample at an applied potential of −0.5V vs Ag/AgCl and illuminated at 532 nm (when “Laser on”). (a) Uncoated sample, the inset image indicates a zoom-in view of the graph to emphasize the photocurrent of the sample (70 nA) and (b) sample coated with a 18 nm $TiO_2$ layer.

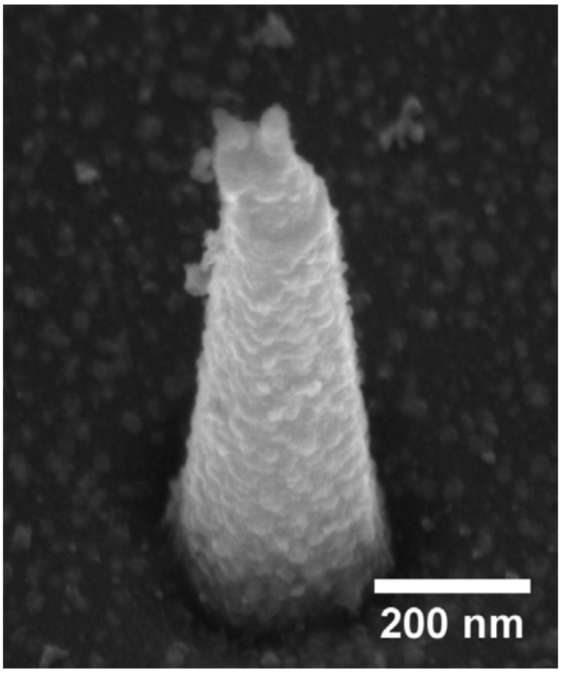


**Figure S5:** SEM image of a silicon nanocone after photo-electrodeposition at 532 nm without $TiO_2$ coating and fully covered with platinum nanoparticles.

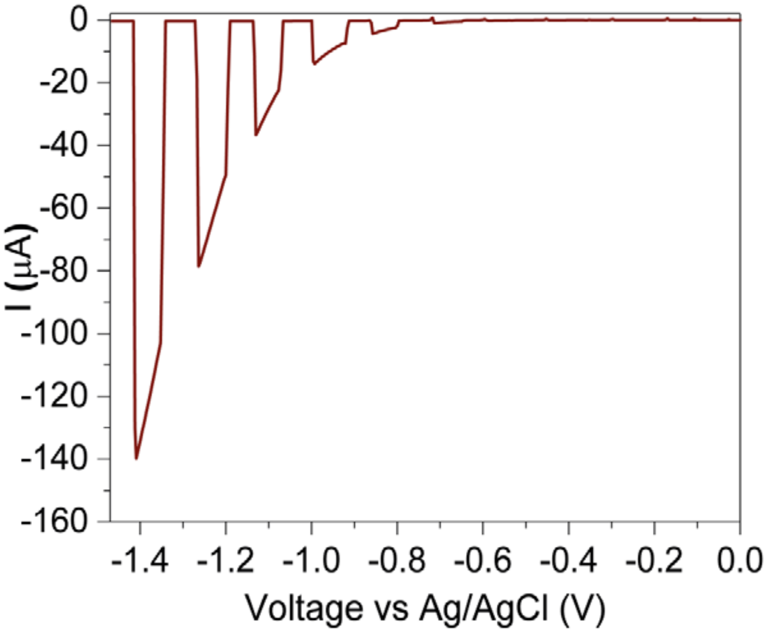


**Figure S6:** Current as a function of the applied potential (vs Ag/AgCl reference electrode) of a silicon nanocone sample, coated with 18 nm $TiO_2$ and excited at 638 nm, with a light beam chopper to show the difference between the dark current and the photocurrent. The excitation took place in presence of $H_2PtCl_6$ (4 mM, pH 11). The scan rate was 50 mV/s. The coverage of this sample with Si nanostructures is different than the one of Figure 3b

and this also explains the much lower photocurrent observed here (ca. 4 μA) at −0.8V. The higher the coverage of the samples, i.e., the higher the amount of nanostructures excited, the higher the photocurrent.

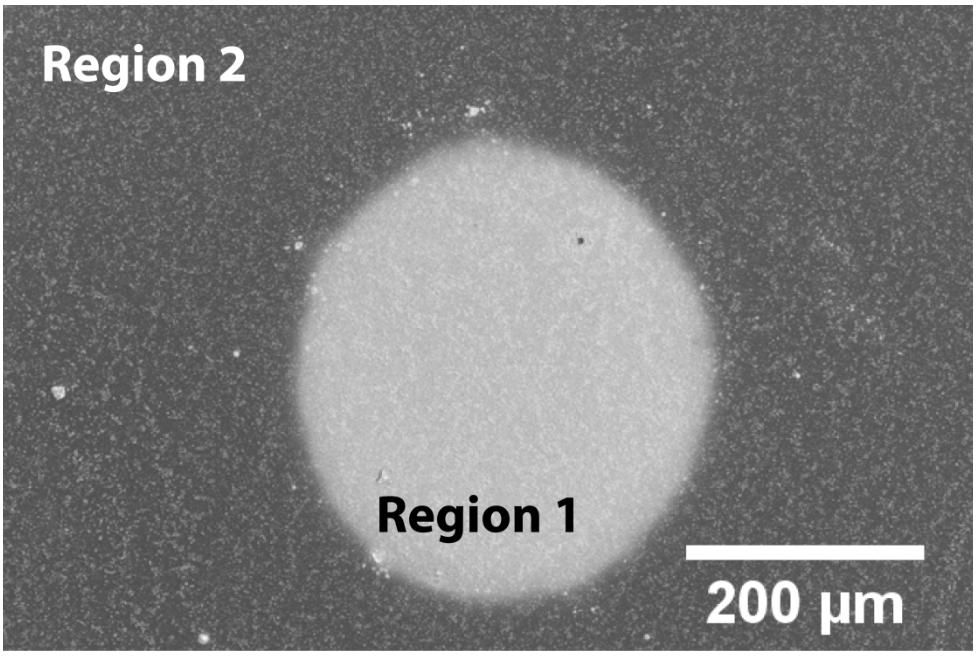


**Figure S7:** SEM image of the illumination spot (region 1; $\lambda_{exc}$ = 532 nm) on a silicon nanostructures sample. Region 2 is outside the illumination area and only small electrodeposited nanoparticles (below 6 nm, see main manuscript text) were identified there. Both regions were in contact with the electrolyte and the bright area corresponds to the diameter of the laser beam as well as to the area where platinum particles are deposited.

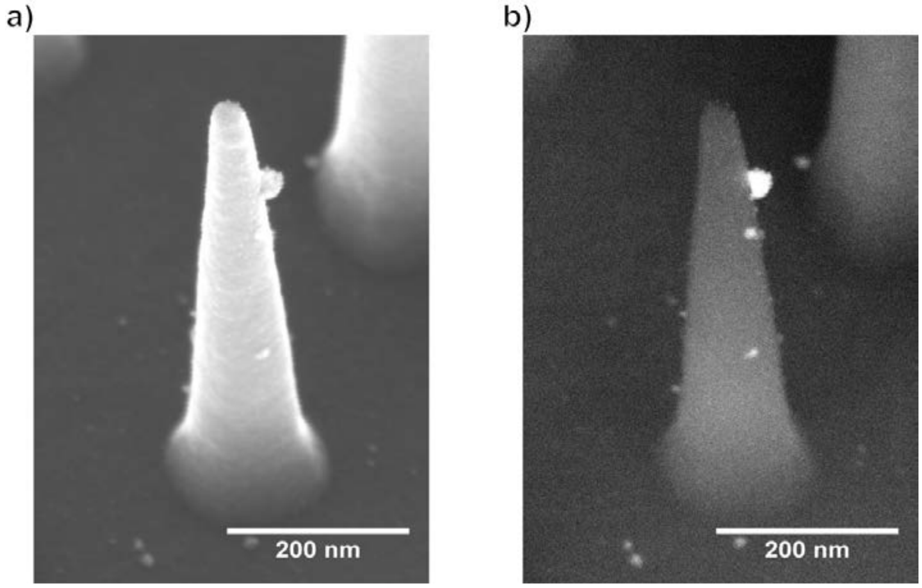


**Figure S8:** (a) Secondary-electron (through-the-lens detector) and (b) backscattered-electron (in-lens mirror detector) SEM images of a silicon nanocone after photo-electrodeposition of platinum in the illuminated region (region 1 in Figure S7).

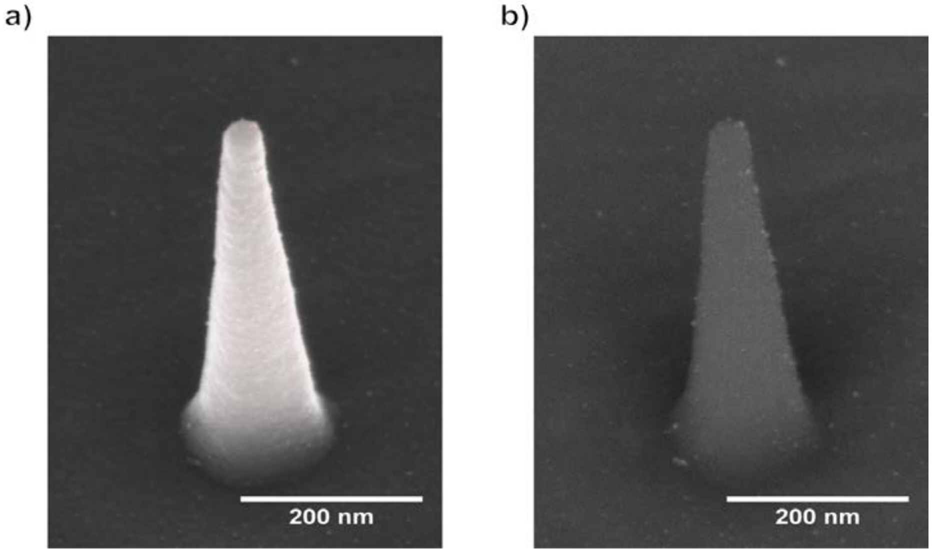


**Figure S9:** (a) Secondary-electron (through-the-lens detector) and (b) backscattered-electron (in-lens mirror detector) SEM images of a silicon nanocone outside the illuminated region. Only few and very small nanoparticles (diameter of ca. 6 nm), which are the result of electrodeposition, are observed outside of the illumination spot (region 2 in Figure S7).

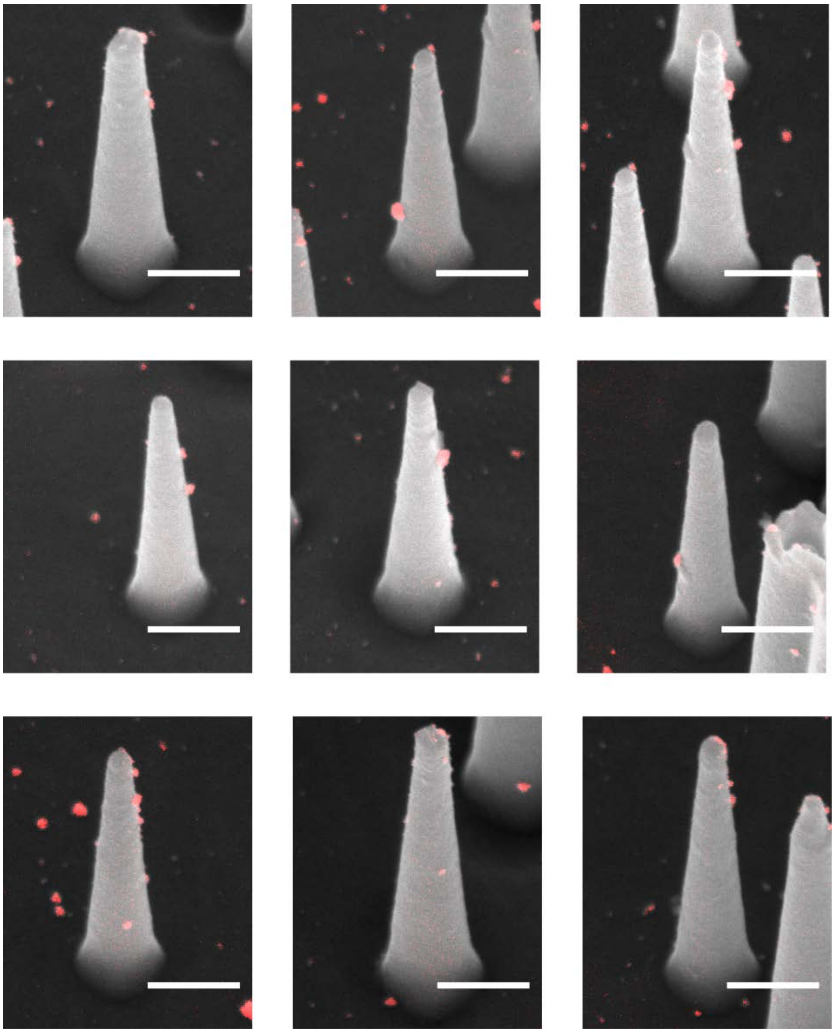

**Figure S10:** Overlays of secondary-electron (grey color; through-the-lens detector) and backscattered-electron (red color; in-lens mirror detector) SEM images of silicon nanocones after photo-electrodeposition of platinum nanoparticles at 532 nm. Scale bar = 200 nm.

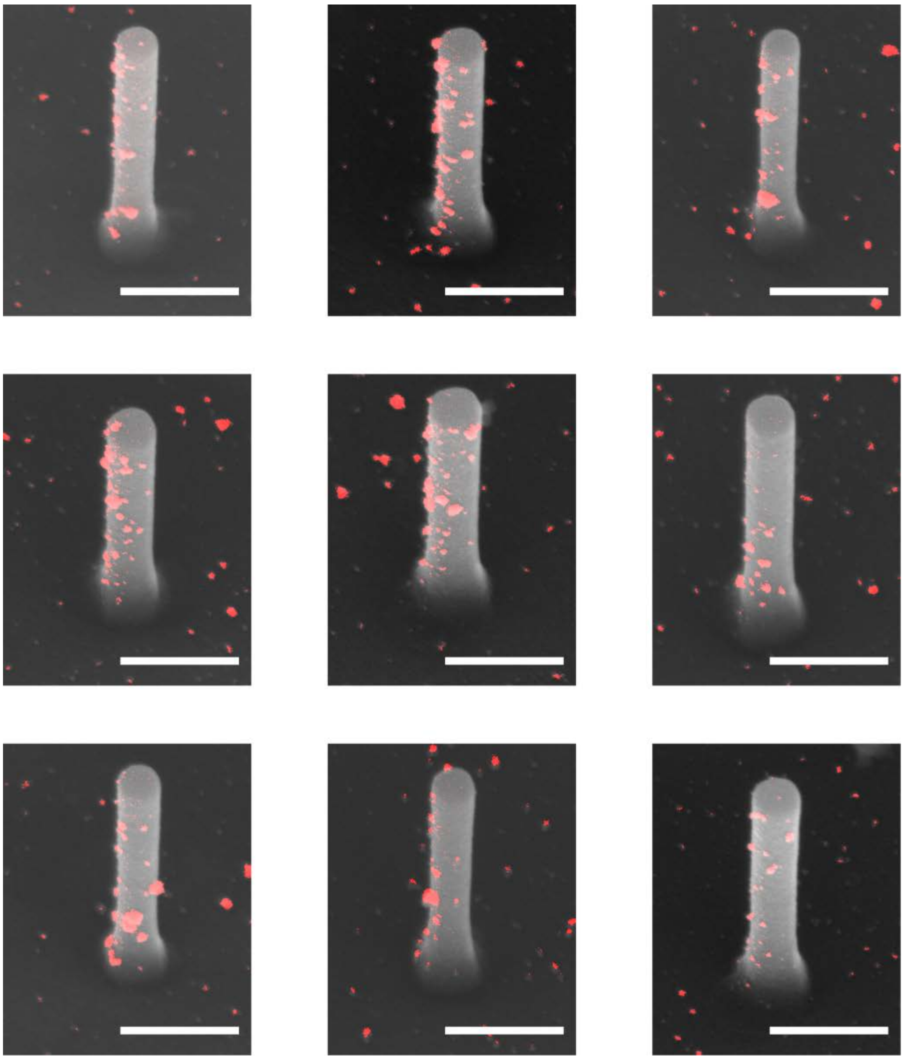

**Figure S11:** Overlays of secondary-electron (grey color; through-the-lens detector) and backscattered-electron (red color; in-lens mirror detector) SEM images of silicon nanowires after photo-electrodeposition of platinum nanoparticles at 532 nm. Scale bar = 200 nm.

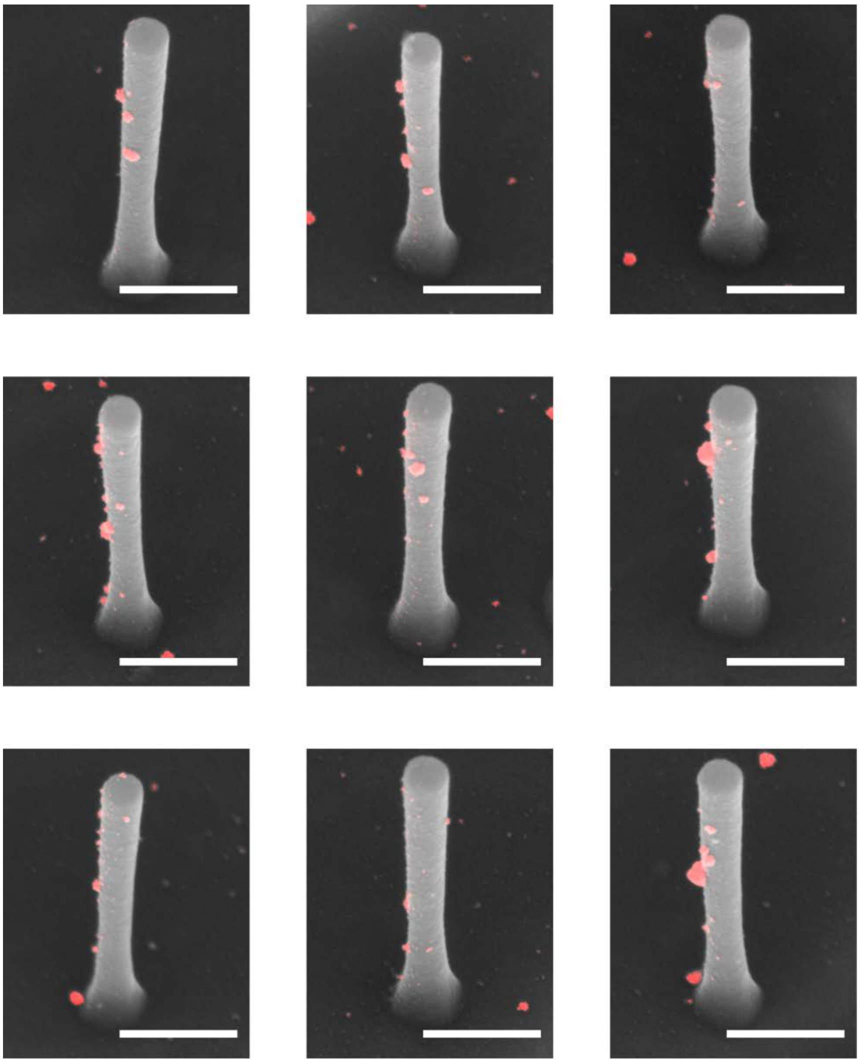

**Figure S12:** Overlays of secondary-electron (grey color; through-the-lens detector) and backscattered-electron (red color; in-lens mirror detector) SEM images of silicon inverted nanocones after photo-electrodeposition of platinum nanoparticles at 532 nm. Scale bar = 200 nm.

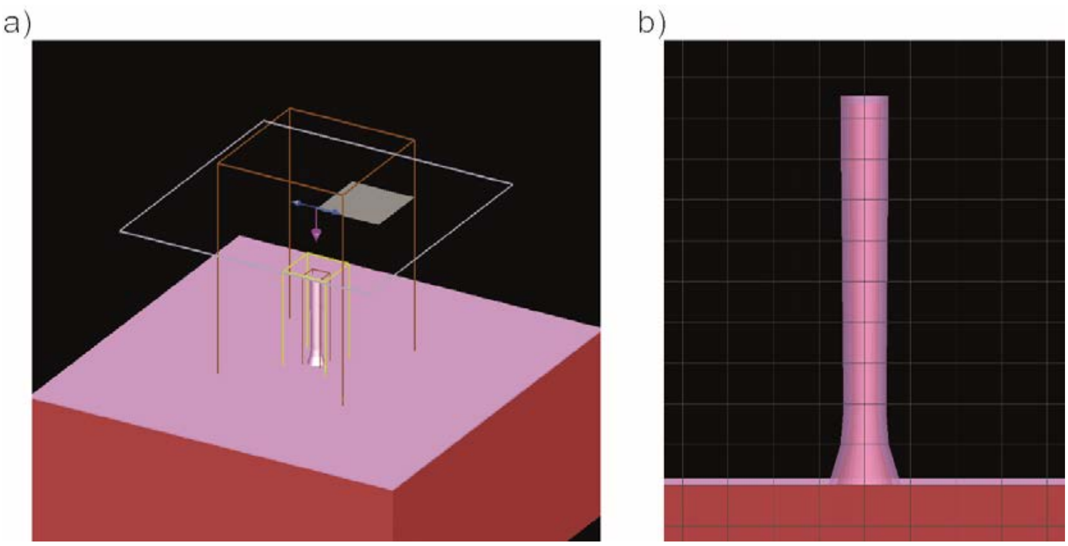


**Figure S13:** (a) Schematic diagram of the FDTD simulations for a silicon nanocone coated with 18 nm $TiO_2$ (shown with pink color) on a silicon substrate excited with a plane wave source (blue/pink arrows). The absorbed power per volume monitor is indicated as a yellow box. (b) Magnification of the simulated silicon nanostructure. The construction of the shape of the inverted nanocone, is the result of a combination of three components (three cones) so as the shape and the dimensions of the structure to correspond to the average shape of the inverted nanocones in the samples.

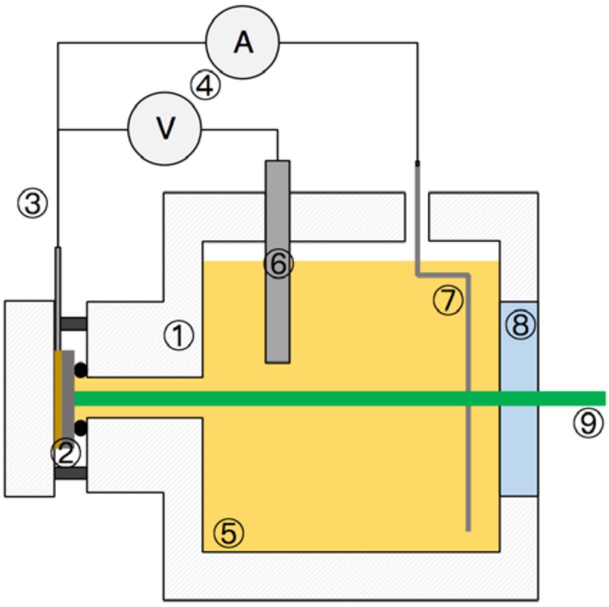


**Figure S14:** Detailed setup for electrodeposition of Pt on silicon nanostructures. 1) Photo-electrochemical cell made of PTFE; 2) sample (silicon) with Au/Cr back contact; 3) electrical connection of the back contact of the sample with the potentiostat and the reference electrode through a conductive aluminum tape; 4) electrical connection of the back contact of the sample with the counter Pt electrode through the potentiostat; 5) electrolyte ($H_2PtCl_6$ in 0.1 M $Na_2SO_4$); 6) reference Ag/AgCl electrode; 7) counter electrode (platinum wire); 8) quartz window; 9) laser beam (532 nm).

# References

1. Siefke, T.; Kroker, S.; Pfeiffer, K.; Puffky, O.; Dietrich, K.; Franta, D.; Ohlídal, I.; Szeghalmi, A.; Kley, E. B.; Tünnermann, A. *Adv. Opt. Mater.* **2016,** *4,* 1780–1786. doi:10.1002/adom.201600250
